# Plasma Fibrinogen at Recurrence as a Prognostic Blood Biomarker in Oral Cavity Squamous Cell Carcinoma

**DOI:** 10.3390/diagnostics16142276

**Published:** 2026-07-21

**Authors:** Rafael Gómez-Fernández, Jorge Vallejo-Díez, Beatriz Zarauza-Santos, Belén Burgos-Vico, Sonia de las Heras-Losada, Luis Miguel Redondo-González

**Affiliations:** Department of Oral and Maxillofacial Surgery, Hospital Universitario Río Hortega, Calle Dulzaina 2, 47012 Valladolid, Spain; jvallejod@saludcastillayleon.es (J.V.-D.); bzarauzas@saludcastillayleon.es (B.Z.-S.); bburgosv@saludcastillayleon.es (B.B.-V.); sdelasheras@saludcastillayleon.es (S.d.l.H.-L.); lredondogo@saludcastillayleon.es (L.M.R.-G.)

**Keywords:** oral squamous cell carcinoma, recurrence, fibrinogen, prognostic biomarkers

## Abstract

**Background:** Preoperative inflammatory biomarkers, particularly the neutrophil-to-lymphocyte ratio (NLR), are established prognostic markers in oral cavity squamous cell carcinoma (OCSCC), yet their relevance at the biologically distinct timepoint of disease recurrence is unknown. We evaluated blood biomarkers measured at recurrence. **Methods:** From a retrospective cohort of 168 surgically treated OCSCC patients, 70 (41.7%) developed recurrence (49 local, 21 distant). NLR, lymphocyte-to-monocyte ratio (LMR), platelet-to-lymphocyte ratio (PLR), plasma fibrinogen, albumin and the prognostic nutritional index (PNI) at relapse were analysed against 5-year overall survival; multivariate logistic regression was adjusted for age and recurrence type. **Results:** Forty-nine of 70 patients (70.0%) died within 5 years. Fibrinogen was higher in deceased patients (median 595 vs. 464 mg/dL; *p* = 0.007) and was the only biomarker to retain independent significance (OR 1.005 per mg/dL; 95% CI 1.000–1.010; *p* = 0.038); in contrast, NLR lost significance (*p* = 0.156). LMR was significant univariately (*p* = 0.036); PLR, albumin and PNI were not. **Conclusions:** In this exploratory single-centre cohort, higher plasma fibrinogen at recurrence was independently associated with 5-year mortality, whereas inflammatory ratios were not. These hypothesis-generating findings warrant validation in larger, multicentre cohorts.

## 1. Introduction

Oral cavity squamous cell carcinoma (OCSCC) is the most frequent malignant tumour of the oral cavity and a major source of cancer-related morbidity and mortality worldwide. Despite advances in surgical resection, reconstructive techniques, and adjuvant radio- and chemotherapy, a substantial proportion of patients develop locoregional or distant recurrence during follow-up, and recurrence remains the principal cause of disease-specific death. Identification of patients at the highest risk of a poor outcome once recurrence has occurred is therefore a clinically relevant and largely unmet need.

Systemic inflammatory biomarkers, particularly the neutrophil-to-lymphocyte ratio (NLR), are established prognostic markers in OCSCC when measured preoperatively [1,2,3,4]. Multiple surgical series and meta-analyses have consistently demonstrated that an elevated preoperative NLR predicts worse survival, with hazard ratios typically ranging from 1.5 to 2.0 [5,6]. These ratios are inexpensive to obtain, are reproducible, and are derived from routine blood tests, which explains their growing use in clinical practice.

However, virtually all available evidence focuses on pretreatment values. The prognostic significance of these biomarkers at the time of tumour recurrence—a critical inflection point characterised by prior treatment exposure, altered tumour biology, and a different host immune and metabolic milieu—has received remarkably little attention [7,8]. Whether the markers that best predict outcome at diagnosis remain the most informative at relapse has, to our knowledge, not been formally examined in this tumour type.

Recent evidence suggests that the relative importance of different host–tumour interaction pathways may shift during disease progression. While systemic inflammation (captured by NLR) may predominate preoperatively, coagulation activation (reflected by fibrinogen) may become more relevant in the context of recurrent or advanced disease. Fibrinogen plays a central role in metastatic progression: it forms protective fibrin shields around circulating tumour cells, facilitates endothelial adhesion, promotes platelet aggregation, and supports angiogenesis [9,10,11]; its synthesis, structure and multifaceted functions in health and disease have recently been reviewed in detail [12]. A comprehensive review by Wu et al. [13] summarised fibrinogen’s role in tumour biology, including its involvement in epithelial–mesenchymal transition and immune evasion.

In OCSCC, fibrinogen measured preoperatively has been associated with prognosis [14,15], and coagulation markers including D-dimer have also been studied [16]; coagulation markers likewise carry prognostic information in related head and neck subsites such as laryngeal squamous cell carcinoma [17]. However, fibrinogen specifically at the time of recurrence has not been investigated in OCSCC.

The present study evaluated blood biomarkers measured at tumour recurrence in a surgically treated OCSCC cohort, with a focus on comparing the relative performance of NLR and fibrinogen at this understudied timepoint. We hypothesised that the prognostic hierarchy at recurrence would differ from the established preoperative paradigm, with coagulation markers gaining prominence over inflammatory ratios.

## 2. Materials and Methods

### 2.1. Study Design and Population

We conducted a retrospective cohort study of consecutive patients diagnosed with primary OCSCC and treated with curative-intent surgery at the Department of Oral and Maxillofacial Surgery of Hospital Universitario Río Hortega (Valladolid, Spain) between 2015 and 2022. This department is a tertiary referral centre that receives patients from ten hospitals across the Castilla y León region. Inclusion criteria were a histological diagnosis of squamous cell carcinoma of the oral cavity and primary surgical treatment with curative intent. Patients with distant metastasis at diagnosis (M1), non-squamous histology, or primary tumours outside the oral cavity were excluded; accordingly, the M component of the TNM classification was not analysed. From the full cohort of 168 patients, 70 (41.7%) developed tumour recurrence during follow-up; these 70 patients constitute the study population of the present analysis.

### 2.2. Staging, Treatment and Recurrence Assessment

Tumours were staged according to the 8th edition of the American Joint Committee on Cancer (AJCC) staging system. Treatment consisted of surgical resection with or without microvascular free-flap reconstruction, followed by adjuvant radiotherapy and/or chemotherapy according to multidisciplinary tumour-board decisions based on pathological risk factors. Recurrence was defined as histologically or radiologically confirmed reappearance of disease after an initial disease-free interval, detected during scheduled clinical and imaging follow-up, and was classified as local (*n* = 49) or distant (*n* = 21). The primary endpoint was 5-year overall survival measured from the date of primary surgery.

### 2.3. Biomarker Assessment at Recurrence

Haematological parameters recorded at the time of recurrence detection included absolute neutrophil, lymphocyte, monocyte and platelet counts (×10^9^/L) obtained from automated complete blood counts, plasma fibrinogen (mg/dL, determined by the Clauss method) and serum albumin (g/dL). The following indices were derived: NLR (neutrophils/lymphocytes), LMR (lymphocytes/monocytes), PLR (platelets/lymphocytes), and the prognostic nutritional index, calculated as PNI = 10 × albumin (g/dL) + 0.005 × lymphocytes (/mm^3^) [18]. Not every parameter was available for every patient, as values depended on the laboratory panels requested in routine clinical practice at relapse; the number of patients with available data therefore ranged from 38 to 54 depending on the parameter, and analyses were performed on an available-case basis.

### 2.4. Statistical Analysis

Continuous variables were expressed as median and interquartile range (IQR), given their non-normal distribution. Comparisons of these variables in survivors and non-survivors were carried out using the Mann–Whitney U test. Categorical variables were summarised as counts and percentages; these were compared using the chi-square or Fisher’s exact test, as appropriate. To identify independent predictors of 5-year mortality, multivariate logistic regression models were constructed, each including age, recurrence type (local vs. distant) and the biomarker of interest; each model therefore contained three predictors, and the biomarkers were not entered simultaneously. For the survival analysis, fibrinogen was additionally analysed as a continuous variable in a Cox proportional-hazards model of overall survival from recurrence. For illustration, patients were also dichotomised at the median value of fibrinogen at recurrence, and overall survival from the time of recurrence was estimated using the Kaplan–Meier method and compared with the log-rank test. A two-sided *p*-value < 0.05 was considered statistically significant. All analyses were performed in Python 3.12 (SciPy 1.11, scikit-learn 1.4, lifelines 0.27).

### 2.5. Ethics

The study was approved by the Research Ethics Committee for Medicinal Products of the Health Areas of Valladolid (CEIm; reference PI-26-197-H, 15 April 2026) and was conducted in accordance with the Declaration of Helsinki. Given the retrospective design and the use of fully anonymised data, the requirement for individual informed consent was waived by the Ethics Committee.

## 3. Results

### 3.1. Baseline Characteristics

The baseline characteristics of the 70 patients with recurrence are summarised in Table 1. The median age was 70 years (IQR 61–80), and the cohort was approximately balanced by sex (37 women, 52.9%; 33 men, 47.1%). The most frequent tumour subsites were the tongue (27.1%), the mandible (18.6%), and the buccal mucosa (17.1%). The cohort was enriched in locally advanced disease: 44.3% of tumours were classified as T4a, and 61.4% of patients had nodal involvement (N+). Most patients received multimodal treatment, with 75.7% undergoing adjuvant radiotherapy with or without chemotherapy, and 60.0% requiring microvascular free-flap reconstruction. Recurrence was local in 49 patients (70.0%) and distant in 21 (30.0%), and the median time from primary surgery to recurrence was 301 days (IQR 153–490). At 5 years, 49 patients (70.0%) had died and 21 (30.0%) were alive.

### 3.2. Biomarkers at Recurrence

Plasma fibrinogen was the strongest prognostic biomarker (Table 2). Median fibrinogen was significantly higher in deceased patients (595 vs. 464 mg/dL; *p* = 0.007), a 28% relative increase. LMR was lower in deceased patients (1.80 vs. 3.25; *p* = 0.036), reflecting relative monocytosis and/or lymphopenia. NLR showed a non-significant trend (5.17 vs. 3.43; *p* = 0.087). Platelets were borderline significant (289 vs. 231 ×10^9^/L; *p* = 0.057). PLR, albumin and PNI were not associated with survival.

### 3.3. Multivariate Analysis

In multivariate logistic regression, fibrinogen was the only biomarker to retain independent significance (OR 1.005 per mg/dL; 95% CI 1.000–1.010; *p* = 0.038), equivalent to an OR of approximately 1.65 per 100 mg/dL increase. NLR did not retain significance (OR 1.063; 95% CI 0.978–1.155; *p* = 0.156). Recurrence type showed a trend but did not reach significance (OR 0.39; 95% CI 0.07–2.18; *p* = 0.286) (Table 3).

### 3.4. Comparison with Preoperative Biomarkers

**Figure 1:**
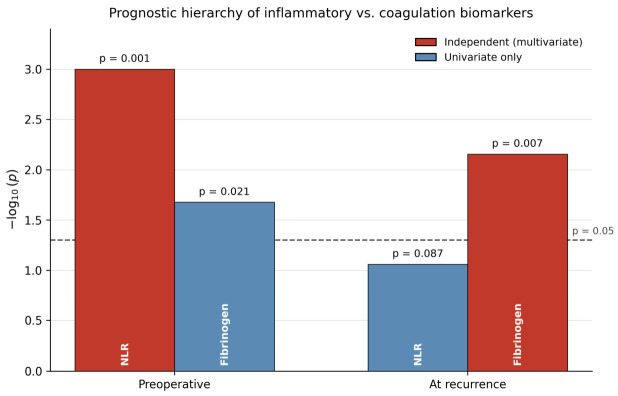
Schematic comparison of the prognostic hierarchy of inflammatory and coagulation biomarkers between the preoperative and recurrence settings in OCSCC. Bars represent −log_10_(*p*) from univariate analysis; red bars indicate independent significance in multivariate analysis. The dashed horizontal line marks the *p* = 0.05 significance threshold. Preoperatively, NLR is the dominant independent predictor (*p* = 0.001); at recurrence, fibrinogen becomes the only independent biomarker (*p* = 0.007), while NLR loses significance (*p* = 0.087).

The prognostic hierarchy at recurrence differs from that observed preoperatively in the cohort. Preoperatively, NLR was the dominant independent prognostic marker (OR 1.33; *p* = 0.025) while fibrinogen showed only univariate significance (*p* = 0.021) without independent prognostic value (*p* = 0.345). At recurrence, this hierarchy is reversed (Figure 1). We hypothesise that this may reflect a change in the predominant host–tumour interaction, although this remains to be confirmed.

### 3.5. Survival Analysis

**Figure 2:**
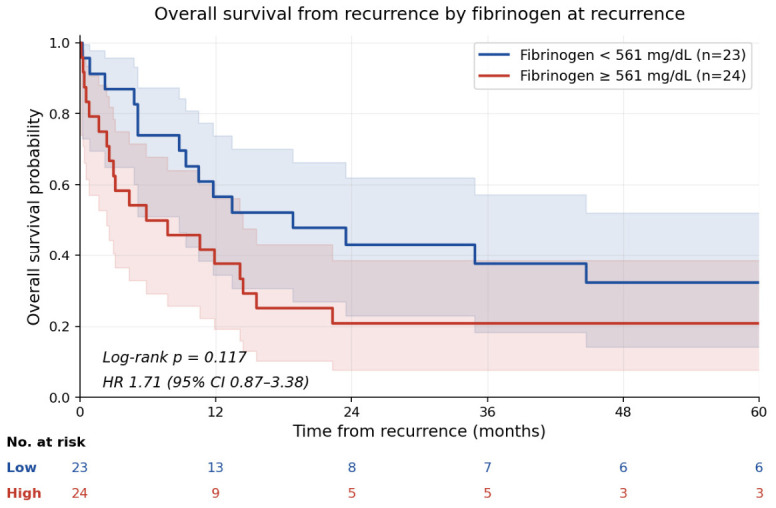
Kaplan–Meier overall survival curves from the time of recurrence, stratified by plasma fibrinogen above versus below the median at recurrence (561 mg/dL). Patients with elevated fibrinogen showed worse overall survival, but this did not reach significance (log-rank *p* = 0.117; hazard ratio 1.71, 95% CI 0.87–3.38). Shaded areas denote 95% confidence bands. Numbers at risk at 0, 12, 24, 36, 48 and 60 months are shown beneath the plot.

Kaplan–Meier analysis by median fibrinogen at recurrence (561 mg/dL) showed worse survival in high-fibrinogen patients that did not reach log-rank significance (*p* = 0.117; HR 1.71, 95% CI 0.87–3.38; Figure 2). Five-year overall survival was 21% in the high-fibrinogen group versus 32% in the low-fibrinogen group. The discrepancy between the non-significant log-rank result and the highly significant continuous-variable analysis (univariate *p* = 0.007; multivariate *p* = 0.038) likely reflects the expected loss of statistical power from dichotomisation in a modest sample. By contrast, a continuous Cox model from recurrence was significant (HR 1.23 per 100 mg/dL, 95% CI 1.04–1.45, *p* = 0.016), consistent with these analyses.

## 4. Discussion

This study reveals a notable shift in the relative prognostic importance of blood biomarkers between pre-treatment and recurrence settings in OCSCC. While preoperative NLR is the dominant independent predictor at diagnosis—as our group and others have demonstrated [1,2,3,4,5,6]—plasma fibrinogen emerges as the biomarker most strongly and independently associated with outcome at recurrence. The studied cohort was characterised by locally advanced, node-positive disease and a high 5-year mortality (70.0%), underlining that recurrent OCSCC represents an aggressive clinical scenario in which refined prognostic stratification is particularly valuable.

The biological interpretation is compelling. At diagnosis, the primary host–tumour interaction is largely immunological: the balance between pro-tumour inflammation (neutrophils) and anti-tumour immunity (lymphocytes) shapes the tumour microenvironment and is captured by NLR [19]. Ruiz-Ranz et al. [3] showed that higher systemic inflammatory markers inversely correlate with stromal lymphocytic infiltration, supporting the interpretation that an elevated NLR mirrors an “immune-cold” phenotype.

At recurrence, the disease biology may be fundamentally different. Recurrent tumour cells have survived primary treatment and may have acquired enhanced capacities for vascular invasion and haemostatic manipulation—processes in which fibrinogen plays a central role [9,10,11]. Fibrinogen facilitates tumour cell adhesion to vascular endothelium, protects circulating tumour cells from immune surveillance, and promotes angiogenesis [9,10]. The predominance of fibrinogen over NLR at recurrence may thus reflect the transition from an immune-dominated to a coagulation-dominated host–tumour interaction.

This “switch” model is supported by data from other tumour types. Hoshino et al. [20] demonstrated that elevated fibrinogen reflects the immunosuppressive tumour microenvironment in oesophageal squamous cell carcinoma, suggesting that fibrinogen may serve as a marker of tumour-induced immune evasion—a mechanism particularly relevant in recurrent disease. Wu et al. [13] further highlighted fibrinogen as a promoter of epithelial–mesenchymal transition, a hallmark of treatment-resistant and recurrent cancers. In OCSCC and other head and neck subsites, preoperative coagulation markers have been linked to outcome [14,15,16,17], but our findings extend this association specifically to the recurrence setting. More broadly, elevated pretreatment fibrinogen is an established adverse prognostic factor across solid tumours, with head and neck among the sites found to show the strongest effect in a large meta-analysis (52 studies, 15,371 patients) [21]. In oral and oropharyngeal cancer specifically, elevated pretreatment fibrinogen has been reported as an independent predictor of both death and relapse (hazard ratio 1.78 for each) [22]; our data extend this association to the recurrence timepoint, although prospective validation is required.

This interpretation is also consistent with the natural history of tumour dissemination. Carcinomas spread predominantly via lymphatic routes in the initial phases, whereas haematogenous dissemination becomes prominent only with increasing tumour burden and established immunosuppression. The concurrent rise in fibrinogen—reflecting endothelial involvement and the haematogenous phase—and the fall in LMR—reflecting immunosuppression and disease progression—in patients who died within five years is coherent with this sequence.

The borderline significance of platelets at recurrence (*p* = 0.057) is consistent with this coagulation-axis model, as platelets interact closely with fibrinogen in the tumour–haemostatic cascade [23]. The significance of LMR (*p* = 0.036) may reflect the role of tumour-associated monocytes and macrophages in promoting immunosuppression and angiogenesis [24]. Taken together, these observations point to a coordinated coagulation–myeloid axis operating at relapse.

An important clinical implication is that standard inflammatory panels (NLR, PLR, LMR) may not be the most informative at recurrence [25]. Fibrinogen, which is routinely measured in coagulation panels and incurs no additional cost, should be considered a key prognostic indicator at this disease stage, and it could be incorporated into the assessment of patients with recurrent OCSCC to refine risk stratification. Furthermore, the prognostic role of fibrinogen at recurrence raises the question of whether anticoagulant or anti-fibrinolytic strategies might have therapeutic potential, as suggested by preclinical data supporting fibrinogen’s role in metastatic progression [9,10]. To our knowledge, this is one of the first studies to systematically evaluate a panel of blood biomarkers at the time of recurrence in OCSCC, revealing a prognostic hierarchy that differs from the established pre-treatment paradigm.

This study has limitations. First, the sample size of patients with available biomarker data at recurrence was modest (38–54 depending on the parameter). This limits statistical power, precludes more complex multivariable modelling, and explains the discrepancy between the continuous and dichotomised survival analyses; the results should therefore be regarded as hypothesis-generating and require validation in larger, preferably multi-institutional cohorts. Second, the retrospective and single-centre design, together with the available-case analysis, may introduce selection and information bias. Third, residual confounding from the type of adjuvant treatment received, the time elapsed from primary surgery, or intercurrent inflammatory or thrombotic conditions cannot be excluded. In particular, the modest number of events (approximately 33 for the fibrinogen model) constrained multivariable adjustment: with each model limited to three predictors to respect the events-per-variable rule, we could not adjust for tumour stage, margin status, perineural or lymphovascular invasion, adjuvant therapy or smoking, so residual confounding remains possible. Moreover, fibrinogen is a non-specific acute-phase reactant, and information on active infection or inflammation at the time of sampling was not recorded; part of its association with overall survival may therefore reflect global host frailty and non-cancer mortality rather than tumour-specific biology. In addition, several biomarkers were screened, so the independent association of fibrinogen should be regarded as exploratory, requiring confirmation in larger cohorts. Finally, no clinical cut-off is proposed; the median split was used only for visualisation, and any future threshold would require external derivation and validation. Prospective studies with standardised biomarker sampling at recurrence are warranted to confirm these findings.

## 5. Conclusions

In this exploratory analysis, higher plasma fibrinogen at tumour recurrence was independently associated with mortality in OCSCC, whereas inflammatory ratios were not so associated. We hypothesise that this may reflect a shift from an inflammation-dominated to a coagulation-associated tumour–host interaction as the disease progresses from primary presentation to recurrence, although this mechanism remains to be demonstrated. These hypothesis-generating findings require external validation before any clinical use, and no cut-off is proposed; fibrinogen at recurrence nonetheless warrants further investigation as a candidate prognostic biomarker.

## Figures and Tables

**Table 1:** Baseline characteristics of the 70 patients with recurrence. Percentages are calculated over the total cohort (n = 70); treatment modality was not recorded for one patient. IQR—interquartile range; AJCC—American Joint Committee on Cancer.

Characteristic	Value
Age, years—median (IQR)	70 (61–80)
Sex—*n* (%)	
Male	33 (47.1)
Female	37 (52.9)
Tumour subsite—*n* (%)	
Tongue	19 (27.1)
Mandible	13 (18.6)
Buccal mucosa	12 (17.1)
Mandibular alveolar crest	8 (11.4)
Floor of mouth	8 (11.4)
Retromolar trigone	4 (5.7)
Maxillary alveolar crest	3 (4.3)
Maxilla	2 (2.9)
Perioral skin	1 (1.4)
T classification (AJCC 8th)—*n* (%)	
T1	10 (14.3)
T2	18 (25.7)
T3	11 (15.7)
T4a	31 (44.3)
N classification (AJCC 8th)—*n* (%)	
N0	27 (38.6)
N1	10 (14.3)
N2a	2 (2.9)
N2b	17 (24.3)
N2c	4 (5.7)
N3b	8 (11.4)
Nx	2 (2.9)
Treatment—*n* (%)	
Surgery alone	13 (18.6)
Surgery + radiotherapy	23 (32.9)
Surgery + chemotherapy	3 (4.3)
Surgery + radiotherapy + chemotherapy	30 (42.9)
Microvascular free-flap reconstruction—*n* (%)	42 (60.0)
Comorbidities and habits—*n* (%)	
Diabetes mellitus	12 (17.1)
Hypertension	31 (44.3)
Cardiovascular disease	19 (27.1)
Tobacco use	25 (35.7)
Alcohol consumption	15 (21.4)
Recurrence—*n* (%)	
Local	49 (70.0)
Distant	21 (30.0)
Time to recurrence, days—median (IQR)	301 (153–490)
5-year status—*n* (%)	
Deceased	49 (70.0)
Alive	21 (30.0)

**Table 2:** Blood biomarkers at recurrence according to 5-year survival status. Values are median (IQR); Mann–Whitney U test. NLR—neutrophil-to-lymphocyte ratio; LMR—lymphocyte-to-monocyte ratio; PLR—platelet-to-lymphocyte ratio; PNI—prognostic nutritional index. * *p* < 0.05.

Biomarker	Alive (*n*)	Median (IQR)	Dead (*n*)	Median (IQR)	*p*
Neutrophils (×10^9^/L)	15	4.60 (3.00–5.90)	39	6.20 (3.90–11.40)	0.071
Lymphocytes (×10^9^/L)	15	1.30 (0.90–2.10)	39	1.21 (0.80–1.60)	0.353
Monocytes (×10^9^/L)	15	0.40 (0.40–0.70)	39	0.60 (0.40–1.10)	0.103
Platelets (×10^9^/L)	14	231 (206–258)	39	289 (213–340)	0.057
Fibrinogen (mg/dL)	14	464 (381–572)	33	595 (514–782)	0.007 *
Albumin (g/dL)	10	3.48 (3.41–3.93)	28	3.50 (3.31–3.82)	0.791
NLR	15	3.43 (1.44–5.25)	39	5.17 (2.37–12.22)	0.087
LMR	15	3.25 (1.38–5.00)	39	1.80 (1.00–2.97)	0.036 *
PLR	14	179 (157–219)	39	222 (147–304)	0.193
PNI	10	42.0 (41.1–47.5)	28	42.2 (37.7–45.8)	0.654

**Table 3:** Multivariate logistic regression for 5-year mortality in patients with recurrence. Two models were used, each adjusted for age and recurrence type, with the corresponding biomarker entered as the variable of interest. OR—odds ratio; CI—confidence interval. * *p* < 0.05.

Variable	OR	95% CI	*p*
Model A—Fibrinogen at recurrence (*n* = 47)			
Age (per year)	1.02	0.98–1.07	0.270
Recurrence type (distant vs. local)	0.39	0.07–2.18	0.286
Fibrinogen at recurrence (per mg/dL)	1.005	1.000–1.010	0.038 *
Model B—NLR at recurrence (*n* = 54)			
Age (per year)	1.02	0.99–1.06	0.198
Recurrence type (distant vs. local)	0.42	0.10–1.81	0.245
NLR at recurrence (per unit)	1.063	0.978–1.155	0.156

## Data Availability

The data presented in this study are available on request from the corresponding author. The data are not publicly available due to privacy restrictions.
